# Increased midgestational IFN-γ, IL-4 and IL-5 in women bearing a child with autism: A case-control study

**DOI:** 10.1186/2040-2392-2-13

**Published:** 2011-08-02

**Authors:** Paula E Goines, Lisa A Croen, Daniel Braunschweig, Cathleen K Yoshida, Judith Grether, Robin Hansen, Martin Kharrazi, Paul Ashwood, Judy Van de Water

**Affiliations:** 1 Division of Rheumatology, Allergy and Clinical Immunology, University of California at Davis, 451 Health Sciences Dr. Suite 6510, Davis, CA 95616, USA; 2 M.I.N.D. Institute, 2825 50th Street, University of California at Davis, Sacramento, CA 95817, USA; 3 Division of Research, Kaiser Permanente, 2000 Broadway, Oakland, CA 94612, USA; 4 Environmental Health Investigations Branch, California Department of Public Health, Richmond, CA 94804, USA; 5 Department of Pediatrics, 2521 Stockton Boulevard, Suite 4100. Sacramento, CA 95817, USA; 6 Genetic Disease Screening Program, California Department of Public Health, Richmond, CA 94804, USA; 7 Department of Medical Microbiology and Immunology, One Shields Ave, University of California at Davis, Davis, CA 95616, USA

## Abstract

**Background:**

Immune anomalies have been documented in individuals with autism spectrum disorders (ASDs) and their family members. It is unknown whether the maternal immune profile during pregnancy is associated with the risk of bearing a child with ASD or other neurodevelopmental disorders.

**Methods:**

Using Luminex technology, levels of 17 cytokines and chemokines were measured in banked serum collected from women at 15 to 19 weeks of gestation who gave birth to a child ultimately diagnosed with (1) ASD (*n *= 84), (2) a developmental delay (DD) but not autism (*n *= 49) or (3) no known developmental disability (general population (GP); *n *= 159). ASD and DD risk associated with maternal cytokine and chemokine levels was estimated by using multivariable logistic regression analysis.

**Results:**

Elevated concentrations of IFN-γ, IL-4 and IL-5 in midgestation maternal serum were significantly associated with a 50% increased risk of ASD, regardless of ASD onset type and the presence of intellectual disability. By contrast, elevated concentrations of IL-2, IL-4 and IL-6 were significantly associated with an increased risk of DD without autism.

**Conclusion:**

The profile of elevated serum IFN-γ, IL-4 and IL-5 was more common in women who gave birth to a child subsequently diagnosed with ASD. An alternative profile of increased IL-2, IL-4 and IL-6 was more common for women who gave birth to a child subsequently diagnosed with DD without autism. Further investigation is needed to characterize the relationship between these divergent maternal immunological phenotypes and to evaluate their effect on neurodevelopment.

## Background

Autism spectrum disorders (ASDs) are a heterogeneous group of neurodevelopmental diseases that manifest in early childhood. Individuals with ASD demonstrate varying degrees of social impairments, deficits in language and communication and stereotypic and repetitive behaviors [1]. There are no clear biological markers for ASD, and current diagnosis relies entirely on behavioral criteria [2,3]. Little is known about the pathology and etiology of the disorders, though genetic, neurologic, environmental and/or immune factors are likely involved [4]. Recent epidemiologic data suggest that approximately 1 in 100 children is diagnosed with an ASD [5,6], highlighting the urgent need for better understanding of this complex disorder.

Evidence has linked various types of maternal immune activation and dysregulation to behavioral disorders, including ASD [7,8]. Mothers of children with ASD have been reported to have a higher incidence of allergic and autoimmune diseases compared to mothers of typically developing children [9-11]. Furthermore, some mothers harbor circulating antibodies that can bind to brain proteins [12-15]. Prenatal immune challenge, such as a bacterial or viral infection, has also been related to behavioral disorders in offspring in both epidemiological studies and animal models [16]. Murine models have shown that exposure to influenza [17], lipopolysaccharide (LPS) [18] and polyinosinic:polycytidylic acid (poly(I:C)) [17,19] during pregnancy results in offspring with altered behavioral phenotypes and brain histopathology, which may be related to aspects of ASD.

The impact of maternal immune activation on the fetal compartment is mediated in part by cytokines and chemokines [7,18,19]. Cytokines and chemokines are proteins that control the intensity, duration and type of immune response. Prenatal exposure to altered levels of cytokines such as IL-2 [20] and IL-6 [21] is sufficient to induce learning disabilities and behavioral changes in murine offspring. Maternal cytokines may affect the fetal compartment directly, as IL-6 has been shown to cross the human placenta (unlike many other cytokines) [22], or indirectly through stimulation of placental cells, which may alter the placental environment and thereby impact the fetus [7].

Few studies have examined midgestational cytokine levels in mothers and ensuing behavioral outcomes in children. We conducted a case-control study using archived maternal blood samples collected during the period from 15 to 19 weeks of gestation to investigate the potential association between serum cytokine profiles and the risk of bearing a child subsequently diagnosed with a neurodevelopmental disorder. We demonstrate the presence of divergent cytokine profiles in serum taken during the second trimester of pregnancy from mothers bearing (1) a child with ASD, (2) a child with a developmental delay (DD) other than ASD or (3) a child from the general population with no known developmental deficiencies (GP).

## Methods

### Subjects

The study sample was based on the Early Markers for Autism (EMA) Study. The EMA Study is a population-based, nested case-control study designed to evaluate biologic markers of susceptibility and exposure in archived maternal midpregnancy and neonatal blood specimens from the same mother-baby pairs. The study subjects are women residing in Orange County, California, who were pregnant in 2000 and 2001 and enrolled in the state's Prenatal Expanded AFP Screening Program [15]. Briefly, three groups were identified: mothers of children with autism spectrum disorder (ASD), mothers of children with DD but not ASD and mothers of GP children. Children with ASD or DD were ascertained from client records obtained from the Regional Center of Orange County. This is one of the 21 regional centers operated by the California Department of Developmental Services (DDS), which are designed to coordinate services for persons with autism and other developmental disabilities. Clients receiving DDS services for ASD or suspected ASD were ascertained as possible subjects for inclusion in this study. Other subjects with moderate to profound developmental disabilities but not ASD (specifically children with an IQ <70 based on standardized tests) were ascertained as other possible DD cases. Diagnoses were confirmed by expert review of all ASD and DD cases as described in the next subsection. GP controls were randomly sampled from the birth certificate files and frequency-matched to ASD cases by sex, birth month and birth year at a 2:1 ratio. All past or current DDS/regional center clients were excluded from the GP population. All study procedures were approved by the institutional review boards of the California Health and Human Services Agency and Kaiser Permanente Northern California.

### Diagnostic verification

After subjects were ascertained from the Regional Center Orange County, ASD and DD diagnoses were verified by trained medical record abstractors following a protocol initially developed by the Metropolitan Atlanta Developmental Disabilities Surveillance Program [23]. Medical record abstractors compiled detailed diagnostic and clinical data from the Regional Center Orange County records for all children initially ascertained as possibly having ASD or DD. Expert clinical review of abstracted data was then conducted by a developmental pediatrician to confirm the ASD or DD diagnoses for this study using *Diagnostic and Statistical Manual of Mental Disorders, Fourth Edition *(DSM-IV) criteria. Children with ASD were further categorized on the basis of disease onset type and cognitive status (presence or absence of intellectual disability (ID) in addition to ASD) using the DSM-IV criteria. Onset type was determined by parental report or clinical observations derived from chart reviews and categorized as "early" (no statement of loss of social and/or language skills, or skill plateau without actual loss), "regressive" (clear loss of previously acquired language and/or social skills) or unable to discern from the record review. The ID determination among ASD subjects was based on composite scores on standardized cognitive and functional tests (with ID: composite score <70; without ID: all scores ≥70 or some scores <70 and others ≥70; unknown: no standardized scores in chart). The final analytic sample consisted of 84 children with ASD (Table 1), 49 children with ID but not ASD (DD) (mild [DSM-IV Text Revision 317] (*n *= 20), moderate [DSM-IV Text Revision 318.0] (*n *= 12), severe [DSM-IV Text Revision 318.1] (*n *= 11), profound [DSM-IV Text Revision 318.2] (*n *= 3), unspecified [DSM-IV Text Revision 319] (*n *= 3), known etiology (*n *= 29) or unknown etiology (*n *= 20)) and 159 GP controls.

**Table 1:** Classification of autism cases in the Early Markers for Autism study^a^

Autism spectrum disorder subgroups	Number of subjects
Total	84
Phenotype	
Autism	55
Asperger's syndrome	0
PDD-NOS	5
Unknown	24
Onset type	
Early onset	64
Regressive	17
Unknown	3
Intellectual disability	
Yes	34
No	30
Unknown	20

^a^PDD-NOS: pervasive developmental disorder not otherwise specified.

### Specimen collection

Maternal midpregnancy serum specimens were retrieved from the Project Baby's Breath prenatal screening specimen archive maintained by the California Genetic Disease Screening Program, at the California Department of Public Health. As part of the screening program, venous blood was collected at 15 to 19 weeks' gestation in serum separator tubes by obstetrical care service providers and underwent expanded α-fetoprotein screening at a single regional laboratory, typically within seven days of collection (median time = 3 days). During transit via the US Postal Service to the regional screening laboratory, no effort was made to control the temperature of the specimens. After testing, leftover specimens were kept under refrigeration for 1 to 2 days and then stored at -20°C. Aliquots of the samples used for this study were stored at -80°C until use with no freeze-thaws prior to testing. All samples were exposed to the same collection and storage protocols.

### Cytokine measurement

Serum concentrations of 17 cytokines and chemokines, including eotaxin, granulocyte macrophage colony-stimulating factor (GM-CSF), IFN-γ, IL-10, IL-12, IL-1β, IL-2, IL-4, IL-5, IL-6, IL-8, IFN-γ-induced protein 10, macrophage inflammatory protein (MIP)-1α, MIP-1β, RANTES and TNF-α were determined using a commercially available multiplex bead-based kit (BioSource Human Bead Kit; Invitrogen, Carlsbad, CA, USA). The assay was carried out in accordance with the protocols provided by the manufacturer. Briefly, 50 μL of serum was incubated with anti-cytokine-conjugated beads in a 96-well filter-bottomed plate on a plate shaker. After two hours, the beads were washed using a vacuum manifold, and biotin-conjugated detection antibodies were added for one-hour incubation. Following a repeat of the washing step, beads were incubated with streptavidin phycoerythrin for 30 minutes. The plates were then read on a Bio-Plex 100 system (Bio-Rad Laboratories, Hercules, CA, USA) and analyzed using Bio-Plex Manager software (Bio-Rad Laboratories) with a five-point standard curve. Reference samples were run on each plate to determine assay consistency.

### Statistical analysis

The distribution of the cytokine concentration values was skewed, and natural log transformation was used to approximate normality. To examine the association of cytokine levels with developmental outcomes after adjustment for possible confounders, we fit separate logistic regression models for ASD vs. GP, ASD vs. DD and DD vs. GP. Case vs. control status was regressed on natural log-transformed cytokine levels with adjustment for several covariates related to the maternal blood draw (maternal weight and gestational age at time of draw) or associated with autism in previous epidemiologic studies (maternal age, race, ethnicity and country of origin). Separate models were run for each cytokine. For all cytokine values that were below the limit of detection (LOD), we assigned a value of LOD/2 prior to log transformation. Fisher's exact tests were used to determine whether there were differences between groups in the proportion of subjects falling within the LOD for each cytokine. Finally, the correlation of individual cytokine levels was tested separately for cases and controls on the basis of the Pearson correlation coefficient.

## Results

A few demographic differences were found between the case and control populations. Compared with the GP controls, the parents of children with ASD were older and the mothers were more likely to be non-Hispanic and born in the United States (Table 2). No differences were observed between the ASD and GP groups with regard to plurality (that is, whether the child was a single birth or one member of a multiple birth), maternal parity (the number of children previously delivered by the mother), maternal weight at blood draw, and child gender (due to matching). Compared to the DD group, children with ASD were more likely to be male and first-born and their mothers were more likely to be older, non-Hispanic and born in the United States (Table 2). Children in the DD group were more likely to be male compared to GP control children (Table 2).

**Table 2:** Characteristics of the Early Markers for Autism study population^a^

Characteristics	ASD (*N *= 84)		DD (*N *= 49)		GP (*N *= 159)		ASD vs. GP	ASD vs. DD	DD vs. GP
	
	*n*	%	*n*	%	*n*	%	*P *value	*P *value	*P *value
Gender							0.91	0.0003	<0.0001
Male	73	86.9	29	59.2	139	87.4			
Female	11	13.1	20	40.8	20	12.6			
Plurality							0.42	1.0	0.34
Singleton	81	96.4	47	95.9	156	98.1			
Multiple	3	3.6	2	4.1	3	1.9			
Parity								0.05	0.24
Primiparous	42	50.0	16	32.7	67	42.1	0.24		
Multiparous	42	50.0	33	67.3	92	57.9			
Mother's race							0.06	0.63	0.62
Caucasian	57	67.9	37	75.5	126	79.2			
Asian	19	22.6	9	18.4	28	17.6			
Other	6	7.1	3	6.1	5	3.1			
Missing	2	2.4	0	0	0	0			
Mother's ethnicity							0.0007	0.0001	0.17
Hispanic	20	23.8	28	57.1	73	45.9			
Non-Hispanic	64	76.2	21	42.9	86	54.1			
Mother's birth country							<0.0001	<0.0001	0.33
United States	45	53.6	16	32.7	71	44.7			
Mexico	9	10.7	22	44.9	58	36.5			
Other	30	35.7	11	22.4	30	18.9			
Mean maternal age, years (±SD)	30.9 (5.2)		28.3 (5.2)		28.2 (5.5)		0.0003	0.006	0.87
Mean paternal age, years (±SD)	34.0 (6.3)		33.0 (7.9)		31.0 (6.5)		0.001	0.41	0.14
Mean last recorded maternal weight prior to blood draw, lb (±SD)	145.1(26.7)		149.1(38.7)		146.9 (33.8)		0.65	0.53	0.56

^a^ASD: autism spectrum disorder; DD: developmental delay; GP: general population.

Cytokine levels measured in maternal serum samples were adjusted for covariates, including gestational age at the time of specimen collection and maternal weight, age, race, ethnicity and country of birth. These adjustments were designed to eliminate variations in cytokine levels related to these factors. Additional file 1 presents the regression results for the potential confounders included in the multivariate models, and Additional file 2 shows the crude unadjusted odds ratios.

**Figure 1:**
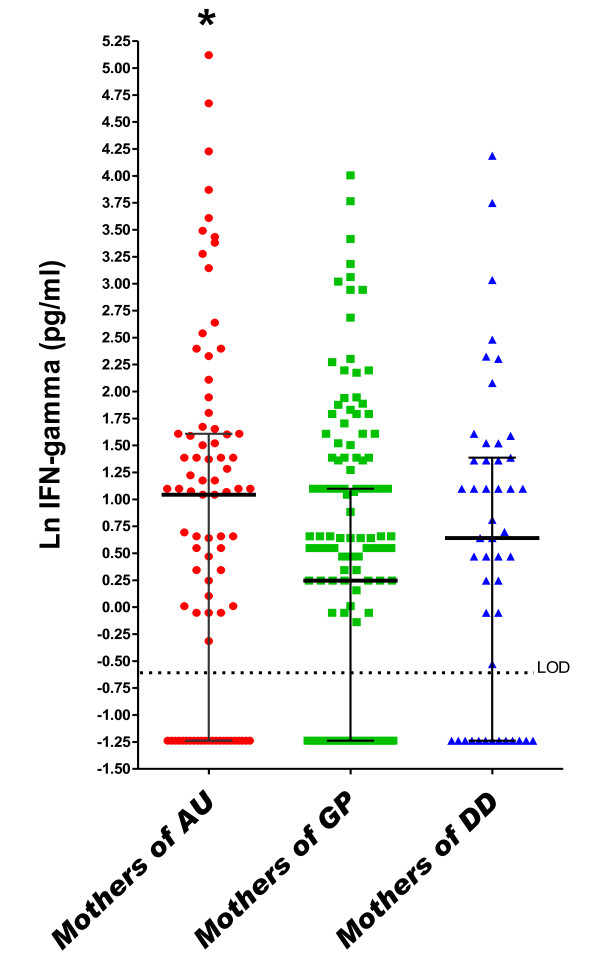
**Midgestational serum IFN-γ**. Scatterplots of natural log-transformed levels of serum IFN-γ in mothers bearing a child with autism spectrum disorder (AU) or a developmental disability other than ASD (DD) compared with a general population control (GP). Each dot represents a single individual. Bars represent the medians and interquartile ranges.

**Figure 2:**
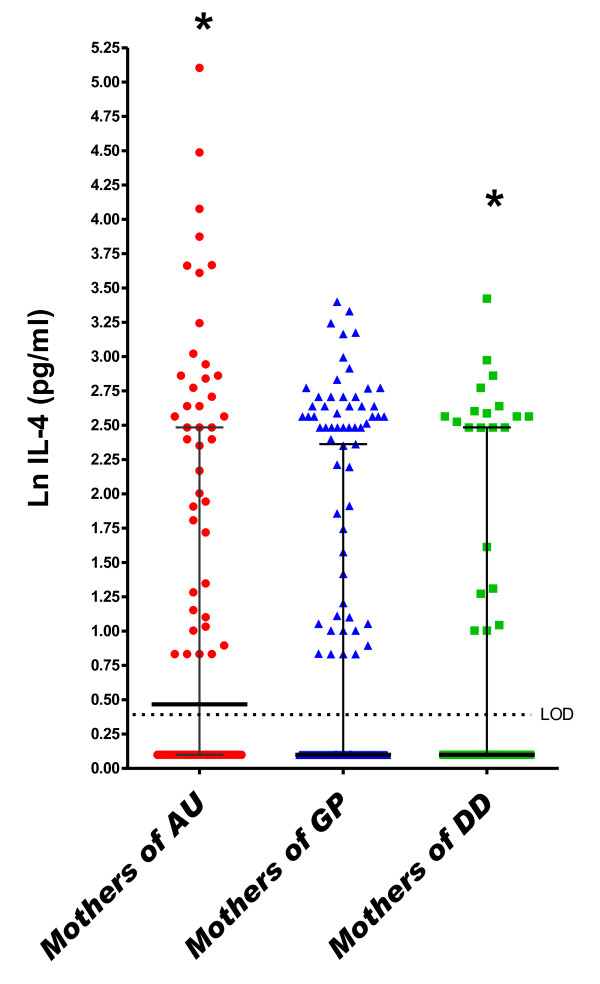
**Midgestational serum IL-4**. Scatterplots of natural log-transformed levels of serum IL-4 in mothers bearing a child with autism spectrum disorder (AU) or a developmental disability other than ASD (DD) compared with a general population control (GP). Each dot represents a single individual. Bars represent the medians and interquartile ranges.

**Figure 3:**
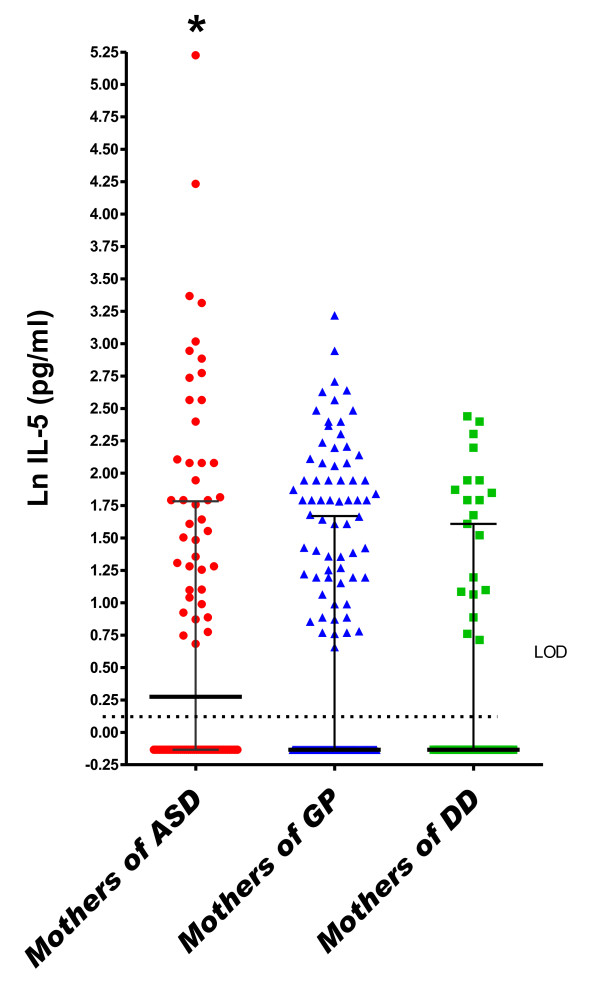
**Midgestational serum IL-5**. Scatterplots of natural log-transformed levels of serum IL-5 in mothers bearing a child with autism spectrum disorder (AU) or a developmental disability other than ASD (DD) compared with a general population control (GP). Each dot represents a single individual. Bars represent the medians and interquartile ranges.

In the logistic regression model adjusted for covariates, a one-unit increase (on the natural log scale) in maternal midpregnancy serum IFN-γ was associated with an approximate 50% increased risk of ASD relative to GP controls (odds ratio (OR) = 1.52, 95% confidence interval (95% CI) = 1.19 to 1.93) (Table 3 and Figure 1). This increase was observed regardless of whether the child had the regressive form (OR_regressive _= 1.77, 95% CI = 1.07 to 2.93) or the early-onset form (OR_early onset _= 1.52, 95% CI = 1.15 to 2.01) of the disorder and regardless of whether ASD occurred in the presence (OR_ID _= 1.56, 95% CI = 1.02 to 2.38) or absence (OR_no ID _= 1.45, 95% CI = 1.04 to 2.03) of ID. Additionally, significantly more subjects in the ASD group were above the LOD for IFN-γ compared to GP subjects (*P *= 0.017 and *P *= 0.06 for the ASD and GP groups, respectively, compared to the DD group) (Additional file 3). The cytokines IL-4 (OR = 1.51, 95% CI = 1.12 to 2.03) and IL-5 (OR = 1.45, 95% CI = 1.07 to 1.98) were similarly associated with an approximate 50% increased risk of ASD relative to GP controls (Table 3 and Figures 2 and 3), regardless of onset type (IL-4: OR_regressive _= 1.86 (95% CI = 1.03 to 3.35), OR_early onset _= 1.47 (95% CI = 1.04 to 2.09), IL-5: OR_regressive _= 1.65 (95% CI = 0.99 to 2.75) and OR_early onset _= 1.52 (95% CI = 1.02 to 2.27)) or the presence of ID (IL-4: OR_ID _= 1.54 (95% CI = 0.94 to 2.52), OR_no ID _= 1.40 (95% CI = 0.93 to 2.10), IL-5: OR_ID _= 1.45 (95% CI = 0.84 to 2.52) and OR_no ID _= 1.50 (95% CI = 0.97 to 2.30)). Higher midpregnancy levels of IFN-γ and IL-5 were also associated with an increased risk of ASD relative to DD controls, although the risk estimates did not achieve statistical significance (Table 3).

**Table 3:** Risk associated with a one-unit increase in the natural log-transformed concentration of cytokines and chemokines measured in midpregnancy maternal serum in the Early Markers for Autism study^a^

	ASD mothers vs. GP mothers		ASD mothers vs. DD mothers		DD mothers vs. GP mothers	
	
Analyte	OR_adj_	95% CI	OR_adj_	95% CI	OR_adj_	95% CI
GM-CSF	1.06	0.88 to 1.28	0.66	0.44 to 1.00	1.28	0.95 to 1.73
IFN-γ	1.52	1.19 to 1.93	1.46	0.94 to 2.26	1.42	0.99 to 2.05
IL-10	1.37	1.00 to 1.87	1.40	0.76 to 2.61	1.65	1.00 to 2.72
IL-12	0.93	0.58 to 1.47	0.39	0.13 to 1.17	0.82	0.42 to 1.62
IL-1β	0.98	0.83 to 1.15	0.84	0.61 to 1.17	1.04	0.81 to 1.32
IL-2	1.22	0.96 to 1.57	1.31	0.77 to 2.21	1.72	1.12 to 2.64
IL-4	1.51	1.12 to 2.03	1.19	0.70 to 2.03	2.18	1.24 to 3.85
IL-5	1.45	1.07 to 1.98	1.70	0.87 to 3.34	1.25	0.72 to 2.18
IL-6	1.10	0.97 to 1.26	0.79	0.62 to 1.02	1.22	1.01 to 1.48
TNF-α	1.07	0.85 to 1.35	0.66	0.42 to 1.04	1.27	0.93 to 1.74
IL-8	0.96	0.80 to 1.15	0.88	0.65 to 1.18	1.17	0.91 to 1.51
Eotaxin	1.18	0.79 to 1.74	0.72	0.38 to 1.37	1.25	0.65 to 2.42
IP-10	1.16	0.74 to 1.81	0.95	0.46 to 1.97	0.70	0.39 to 1.27
MCP-1	1.06	0.75 to 1.50	0.83	0.45 to 1.53	1.15	0.75 to 1.77
MIP-1α	1.11	0.94 to 1.31	0.87	0.61 to 1.23	1.10	0.86 to 1.41
MIP-1β	1.10	0.93 to 1.30	0.79	0.53 to 1.17	1.16	0.89 to 1.52
RANTES	0.92	0.65 to 1.31	0.97	0.44 to 2.14	0.98	0.55 to 1.76

^a^ASD: autism spectrum disorder; GP: general population; DD: developmental delay; OR_adj_: adjusted odds ratio; 95% CI: 95% confidence interval; GM-CSF: granulocyte macrophage colony-stimulating factor; MIP-1: macrophage inflammatory protein; MCP-1: monocyte chemotactic protein-1; IP-10: IFN-γ-induced protein 10; RANTES: regulated upon activation, normal T cell expressed and secreted. Results are adjusted for maternal weight and gestational age of the fetus at the time of specimen collection, as well as for maternal age, race, ethnicity and country of origin.

**Figure 4:**
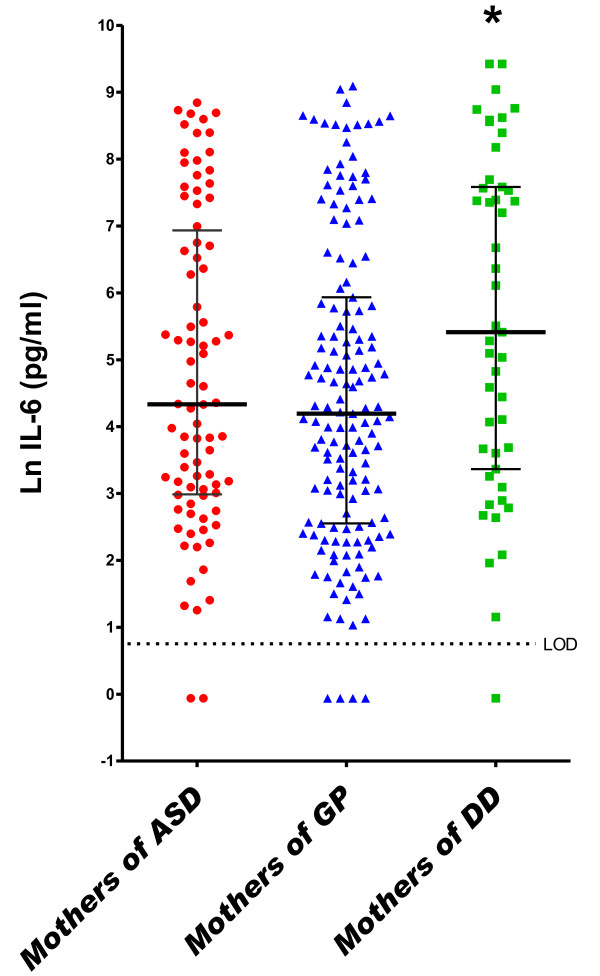
**Midgestational serum IL-6**. Scatterplots of natural log-transformed levels of serum IL-6 in mothers bearing a child with autism spectrum disorder (AU) or a developmental disability other than ASD (DD) compared with a general population control (GP). Each dot represents a single individual. Bars represent the medians and interquartile ranges.

**Figure 5:**
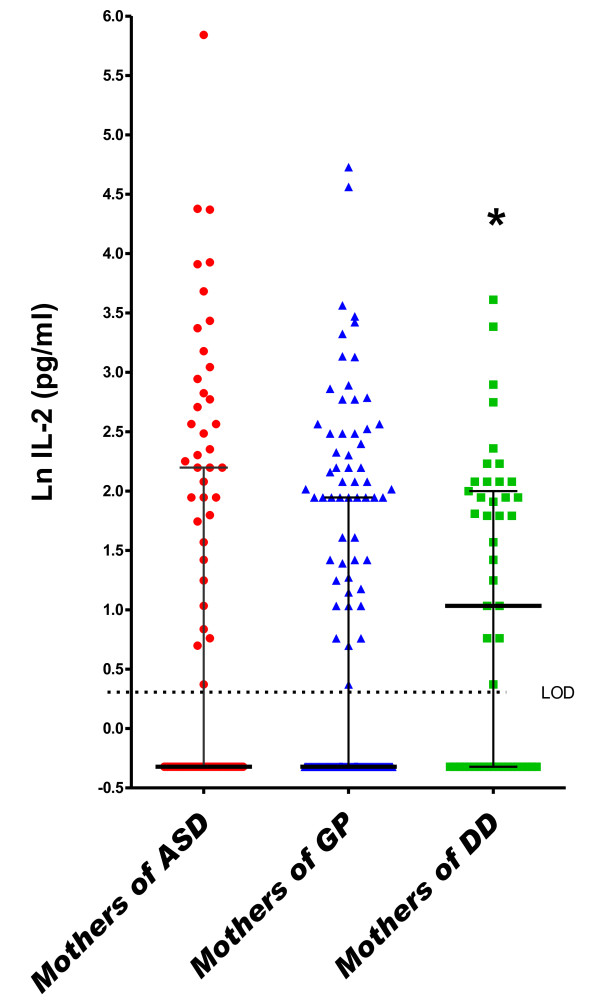
**Midgestational serum IL-2**. Scatterplots of natural log-transformed levels of serum IL-2 in mothers bearing a child with autism spectrum disorder (AU) or a developmental disability other than ASD (DD) compared with a general population control (GP). Each dot represents a single individual. Bars represent the medians and interquartile ranges.

In the DD group, a one-unit increase (on the natural log scale) in midpregnancy serum concentrations of IL-2 (OR = 1.72, 95% CI = 1.12 to 2.64), IL-4 (OR = 2.18, 95% CI = 1.24 to 3.85) and IL-6 (OR = 1.22, 95% CI = 1.01 to 1.48) was associated with increased risk of DD relative to GP controls in adjusted analyses (Table 3 and Figures 2, 4 and 5). Finally, the risk for both ASD and DD increased with increasing levels of IL-10 relative to GP controls, but risk estimates were of borderline significance (Table 3).

Finally, Pearson correlation coefficients were used to determine which cytokines were elevated or reduced together in each group. This was specifically of interest, given that IFN-γ and IL-4 and IL-5, commonly thought to be counterregulatory cytokines, were elevated in mothers bearing a child with ASD. This analysis was designed to determine whether these cytokines were elevated in the same individuals or whether they represented two subgroups: one with high levels of IFN-γ and one with high levels of IL-4 and IL-5. Maternal serum IFN-γ, IL-4 and IL-5 levels were significantly correlated in the ASD group (regardless of onset type and cognitive status), demonstrating that IFN-γ was indeed elevated alongside IL-4 and IL-5. These cytokines were also significantly correlated in the GP group, although the correlation between IFN-γ and IL-4 or IL-5 was not as strong as in the ASD group (Table 4). While IL-4 was significantly correlated with IL-5 in the DD group, there was no correlation between maternal serum IFN-γ and either IL-4 or IL-5 in this group (Table 4). IL-4 was also significantly positively correlated with IL-2 in all groups and with IL-6 in the ASD early-onset and ASD without ID subgroups. Finally, IL-6 and IL-2 were positively correlated in the GP group only (Table 4). The differences across study groups in the strength of the correlations may be partly explained by the underlying structure of the data (that is, the large percentage of concentrations below the LOD for some cytokines) and thus should be interpreted with caution.

**Table 4:** Pearson correlation coefficients of cytokines measured in maternal midpregnancy serum in the Early Markers for Autism study^a^

Measurements	ASD (*N *= 84	ASD regression (*N *= 17)	Early-onset ASD (*N *= 64)	ASD with ID (*N *= 34)	ASD without ID (*N *= 30)	DD (*N *= 49)	GP (*N *= 159)
Cytokines elevated in ASD							
IFN-γ and IL-4	0.95**	0.95**	0.81**	0.98**	0.97**	0.05	0.48**
IFN-γ and IL-5	0.73**	0.97**	0.55**	0.91**	0.87**	-0.07	0.38**
IL-4 and IL-5	0.72**	0.89**	0.83**	0.97**	0.82**	0.41**	0.77**
Cytokines elevated in DD							
IL-2 and IL-4	0.87**	0.72**	0.62**	0.97**	0.78**	0.57**	0.43**
IL-2 and IL-6	0.02	0.34	0.18	-0.06	0.34	-0.15	0.24**
IL-4 and IL-6	0.15	0.46	0.34*	-0.09	0.42*	0.13	0.11

^a^ASD: autism spectrum disorder; ID: intellectual disability; DD: developmental delay; GP: general population. **P  < 0.0001, *P  < 0.05.

## Discussion

In the present study, we characterized levels of cytokines and chemokines in archived maternal serum collected during midpregnancy and analyzed whether these levels were related to ASD and DD outcomes in the child. We have provided evidence for increased IL-4, IL-5 and IFN-γ in mothers bearing a child with ASD. In contrast, mothers bearing a child with DD but not ASD demonstrated increased levels of the cytokines IL-2, IL-4, IL-6 and GM-CSF as well as the chemokine MIP-1α. These contrasting immune profiles described in the ASD and DD groups indicate that different maternal immune profiles during pregnancy may be linked to divergent neurodevelopmental outcomes in the child. The results from both the ASD and DD groups suggest possible elevation in IL-10 relative to the GP controls. This finding is interesting, given that IL-10 is an immunomodulatory cytokine that may be expressed to counteract the effects of inflammatory cytokines.

### Maternal immune activity downregulated during pregnancy

The maternal immune system is uniquely regulated during pregnancy to optimize the gestational environment of the fetus [24]. Primarily, it must be poised to protect the mother and fetus from pathogens and other potentially harmful environmental factors. Simultaneously, robust maternal cellular immune responses must be suppressed to avoid rejection of the fetus as a foreign allograft [25]. Evidence suggests that under normal circumstances, pregnancy shifts the maternal immune system toward a more tolerant, low inflammatory state that involves decreased production of cytokines such as IL-6 and IFN-γ and increased production of the more regulatory cytokines, including IL-4, IL-5 and IL-10 [26-29]. Mothers of children with ASD and DD demonstrated increased levels of the inflammatory cytokines IFN-γ and IL-6, respectively, which may be indicative of an atypical immune state during gestation.

### Proper maternal immune regulation is important for healthy fetal development

Dramatic changes in maternal immune homeostasis during pregnancy (in response to infection, disease or other environmental influences) are associated with complications such as miscarriage, preterm delivery and preeclampsia [30]. Maternal immune responses can also affect the development of the fetal nervous system [31,32]. Epidemiological studies have suggested that prenatal infections may be related to neurological disorders such as schizophrenia and autism [33,34]. Furthermore, animal models have repeatedly demonstrated that robust maternal immune responses during pregnancy can alter offspring behavior and brain histopathology [17,19,35-37]. Various cytokines, including IL-6 and IL-2, have been shown to mediate some of these effects [18,20,21]. We suggest that atypical maternal immune function during pregnancy may be related to ASD or DD outcomes among children.

### *Mothers bearing a child with ASD had increased levels of IFN-*γ, *IL-4 and IL-5*

In the current study, mothers bearing a child with ASD had significantly increased levels of serum IFN-γ, IL-4 and IL-5. IFN-γ, the most dramatically elevated cytokine in this population, is involved in aspects of defense against intracellular pathogens, tumor surveillance, autoimmunity, allergy and pregnancy. Peripheral IFN-γ levels are low in healthy pregnancies, and increased production of peripheral IFN-γ is often related to complications such as preeclampsia [38]. IFN-γ is produced by a subset of activated T cells, though its primary source is natural killer (NK) cells. During pregnancy, a unique population of IFN-γ-producing NK cells accumulates in the uterus, where they have a vital role in placental development [39]. The increased levels of serum IFN-γ observed in mothers bearing a child with ASD may be indicative of imbalanced immune function at the maternal-fetal interface, which could lead to improper placental formation and thereby incomplete support of fetal development. Alternatively, increased serum IFN-γ may be due to peripheral immune activity, including a response to infection, or to an immune-mediated disorder. Interestingly, a recent examination of archived neonatal blood spots from children with ASD revealed no elevation in pathogen-specific immunoglobulin G (IgG) levels relative to controls, suggesting that prenatal infection may not be involved [40].

Epidemiological data previously reported by our group indicated a higher prevalence of allergy and asthma during pregnancy in mothers of children with ASD [11]. That 2005 study examined physician-diagnosed medical conditions in over 2,500 women enrolled in the Kaiser Permanente Medical Care Program. Interestingly, the midpregnancy cytokine profile we describe in the ASD group in the present study (increased IL-4, IL-5 and IFN-γ) may be consistent with an allergic asthma clinical phenotype [41-44]. While IL-4 and IL-5 are known to be upregulated in allergic asthma, IFN-γ is generally thought to be downregulated [45]. However, several reports have shown increased production of IFN-γ in addition to IL-4 and IL-5 in allergic asthma [41-44]. This has also been observed during pregnancy in women with asthma, when higher levels of IFN-γ correlated with worsening maternal and fetal health [43]. Future studies should address the impact of prenatal allergy and asthma on fetal neurodevelopment and further explore the possible connection to behavioral disorders.

### Alternative cytokine profile in mothers bearing a child with a developmental delay

We noted that mothers bearing a child with DD but not ASD demonstrated a different midgestational immune profile. The risk of DD was associated with higher levels of the cytokines IL-2, IL-4 and IL-6. Interestingly, IL-6 is part of a cytokine family with well-defined neurological impacts [46]. Extensive evidence links prenatal immune responses involving increased production of inflammatory cytokines such as IL-6 to pregnancy complications and neurological abnormalities among offspring [30,47]. Mouse models have shown that prenatal exposure to IL-6 or mimics of infectious agents such as LPS or poly(I:C) can induce behavioral changes and brain abnormalities among offspring [18,19,21,48]. Similarly, prenatal exposure to high levels of IL-2 has been shown to induce behavioral differences in murine models [20]. On the basis of these animal studies, it has been suggested that prenatal exposure to these inflammatory conditions may be relevant to the development of autism [20,48]. However, our results showed elevated IL-6 and IL-2 in mothers bearing a child with DD but not autism. Therefore, we propose that elevated levels of these cytokines have a global effect on neurodevelopment, resulting in cognitive impairment but not necessarily autism.

### Maternal cytokines and fetal neurodevelopment

The mechanism by which maternal cytokines affect fetal neurodevelopment is unclear, though the central nervous system (CNS) and immune system interact extensively during fetal development and throughout life. Neuroimmune cross-talk is facilitated by shared signaling pathways and commingling of cellular and soluble components from each system [49,50]. Evidence suggests that immune components such as cytokines can affect aspects of neurogenesis, neuronal migration and synaptic plasticity, depending on the timing and level of exposure [46,51,52]. The developing CNS is especially vulnerable to immunological and environmental influences because the fetus has an immature blood-brain barrier and limited capacity for detoxification and excretion [53]. Under normal circumstances, the placenta forms a barrier between the maternal and fetal circulation, though some maternal immune factors, including IgG and IL-6, are permitted to cross the placenta [22,54,55]. When passage of maternal immune components is blocked, the placenta may respond to entities at the maternal-fetal interface and alter the fetal compartment [56]. For example, IFN-γ is not known to pass between the maternal and fetal circulation, though IFN-γ and its receptors are expressed by maternal and fetal cells at the maternal-fetal interface [57]. Therefore, maternal immune components can interact with fetal development both directly and indirectly. The specific neurodevelopmental impact of the different cytokine profiles observed in the present study remains to be determined.

### Study limitations

Although our study provides valuable, temporally relevant information regarding prenatal immune status and the child's developmental outcome, a few primary limitations must be considered. First, immune activation in the peripheral blood is not necessarily representative of immune activity at the maternal-fetal interface. Examination of more spatially relevant immune parameters would require placental or amniotic specimens, which were not available in this study. Despite this limitation, the archived serum samples examined provide valuable insight into global maternal immune status during a developmentally relevant window. Second, this study is cross-sectional, as the serum specimens represent a single time point between 15 and 19 weeks of gestation. Maternal immune activity is likely to change throughout pregnancy, and the gestational immune environment outside 15 to 19 weeks' gestation is also developmentally relevant. Future longitudinal studies will provide a more complete picture of the relationship between maternal immune activation throughout pregnancy and fetal neurodevelopment. Third, data regarding the occurrence of infection, allergy and asthma were not available for the population included in this study, so the factors underlying the observed cytokine profiles are unknown. Replication studies are required to further verify the findings described herein. Fourth, it should be noted that the study groups were matched on the basis of child characteristics rather than maternal characteristics. However, two of the three offspring characteristics used for matching, birth month and birth year, relate directly to an important characteristic of the mother (that is, season during midpregnancy) that may influence cytokine levels through their association with seasonal illness. While the remaining covariates were adjusted for in multivariable logistic regression analysis, there is a possibility that our results could be biased because of residual confounding. Finally, diagnoses were made on the basis of medical record abstraction rather than via direct assessment. While we are confident in the consistency and accuracy of our expert medical record review, we recognize that this approach is likely to introduce some level of error, in part because of the differences in the amount and specificity of documentation in the medical records. Our future analyses will involve direct observation and diagnosis of subjects.

## Conclusions

In conclusion, we describe different midgestational immune profiles in mothers bearing children with ASD and mothers bearing children with DD. Mothers bearing children with autism had cytokine profiles that may be consistent with an allergy and/or asthma immune phenotype, while mothers bearing children with DD but not autism demonstrated a more inflammatory phenotype. Cytokines and other immune components are known to affect the health of pregnancy and can influence fetal neurodevelopment. The possibility that divergent maternal immune profiles during pregnancy have different effects on fetal neurodevelopment warrants further investigation.

## Abbreviations

ASD: autism spectrum disorder; CNS: central nervous system; DD: developmental delay; DDS: Department of Developmental Services; EMA: Early Markers for Autism; GM-CSF: granulocyte macrophage colony-stimulating factor; GP: general population; IFN: interferon; IL: interleukin; LOD: limit of detection; LPS: lipopolysaccharide; MIP: macrophage inflammatory protein; RC: regional center; NK: natural killer.

## Competing interests

The author declares that they have no competing interests.

## Authors' contributions

PEG carried out the Luminex assays, managed raw data, interpreted the findings and was the primary writer of the manuscript. LAC contributed to the conception and design of the study, obtaining funding, acquiring data, interpreting the data and preparing the manuscript. DB carried out the Luminex assays and edited the manuscript. CKY conducted statistical analyses, interpreted the data and edited the manuscript. JG contributed to the conception and design of the study, the analysis and interpretation of the data and critical revision of the manuscript. RH and MK contributed to the acquisition of data, the interpretation of the data and critical revision of the manuscript. PA contributed to the study design, the interpretation of data and critical revision of the manuscript. JVdW contributed to the intellectual design of the study and the interpretation of data and edited the manuscript. All authors read and approved the final manuscript.

## Supplementary Material

Additional file 1 **Adjusted odds ratios for covariates**. Adjusted odds ratios for each covariate analyzed in the subject population for selected analytes from Table 3. Click here for file

Additional file 2 **Crude odds ratios**. Crude unadjusted odds ratios for all analytes in Table 3. Click here for file

Additional file 3 **Limit of detection**. Numbers and percentages of subjects found to be below the limit of detection (<LOD) for each analyte across groups. Click here for file

## References

[B1] American Psychiatric Association Diagnostic and Statistical Manual of Mental Disorders 1994 4 Washington, DC: American Psychiatric Association

[B2] Lord C Pickles A McLennan J Rutter M Bregman J Folstein S Fombonne E Leboyer M Minshew N Diagnosing autism: analyses of data from the Autism Diagnostic Interview J Autism Dev Disord 1997 27 501 517 10.1023/A:1025873925661 9403369

[B3] Lord C Risi S Lambrecht L Cook EH Jr Leventhal BL DiLavore PC Pickles A Rutter M The autism diagnostic observation schedule-generic: a standard measure of social and communication deficits associated with the spectrum of autism J Autism Dev Disord 2000 30 205 223 10.1023/A:1005592401947 11055457

[B4] Pardo CA Eberhart CG The neurobiology of autism Brain Pathol 2007 17 434 447 10.1111/j.1750-3639.2007.00102.x 17919129 PMC8095519

[B5] Kogan MD Blumberg SJ Schieve LA Boyle CA Perrin JM Ghandour RM Singh GK Strickland BB Trevathan E van Dyck PC Prevalence of parent-reported diagnosis of autism spectrum disorder among children in the US, 2007 Pediatrics 2009 124 1395 1403 10.1542/peds.2009-1522 19805460

[B6] Autism and Developmental Disabilities Monitoring Network Surveillance Year 2006 Principal Investigators Centers for Disease Control and Prevention (CDC) Prevalence of autism spectrum disorders: Autism and Developmental Disabilities Monitoring Network, United States, 2006 MMWR Surveill Summ 2009 58 1 20 20023608

[B7] Jonakait GM The effects of maternal inflammation on neuronal development: possible mechanisms Int J Dev Neurosci 2007 25 415 425 10.1016/j.ijdevneu.2007.08.017 17949937

[B8] Ashwood P Wills S Van de Water J The immune response in autism: a new frontier for autism research J Leukoc Biol 2006 80 1 15 10.1189/jlb.1205707 16698940

[B9] Money J Bobrow NA Clarke FC Autism and autoimmune disease: a family study J Autism Child Schizophr 1971 1 146 160 10.1007/BF01537954 5172389

[B10] Sweeten TL Bowyer SL Posey DJ Halberstadt GM McDougle CJ Increased prevalence of familial autoimmunity in probands with pervasive developmental disorders Pediatrics 2003 112 e420 10.1542/peds.112.5.e420 14595086

[B11] Croen LA Grether JK Yoshida CK Odouli R Van de Water J Maternal autoimmune diseases, asthma and allergies, and childhood autism spectrum disorders: a case-control study Arch Pediatr Adolesc Med 2005 159 151 157 10.1001/archpedi.159.2.151 15699309

[B12] Martin LA Ashwood P Braunschweig D Cabanlit M Van de Water J Amaral DG Stereotypies and hyperactivity in rhesus monkeys exposed to IgG from mothers of children with autism Brain Behav Immun 2008 22 806 816 10.1016/j.bbi.2007.12.007 18262386 PMC3779644

[B13] Singer HS Morris CM Gause CD Gillin PK Crawford S Zimmerman AW Antibodies against fetal brain in sera of mothers with autistic children J Neuroimmunol 2008 194 165 172 10.1016/j.jneuroim.2007.11.004 18093664

[B14] Zimmerman AW Connors SL Matteson KJ Lee LC Singer HS Castaneda JA Pearce DA Maternal antibrain antibodies in autism Brain Behav Immun 2007 21 351 357 10.1016/j.bbi.2006.08.005 17029701

[B15] Croen LA Braunschweig D Haapanen L Yoshida CK Fireman B Grether JK Kharrazi M Hansen RL Ashwood P Van de Water J Maternal mid-pregnancy autoantibodies to fetal brain protein: the early markers for autism study Biol Psychiatry 2008 64 583 588 10.1016/j.biopsych.2008.05.006 18571628 PMC2574992

[B16] Meyer U Feldon J Schedlowski M Yee BK Towards an immuno-precipitated neurodevelopmental animal model of schizophrenia Neurosci Biobehav Rev 2005 29 913 947 10.1016/j.neubiorev.2004.10.012 15964075

[B17] Shi L Fatemi SH Sidwell RW Patterson PH Maternal influenza infection causes marked behavioral and pharmacological changes in the offspring J Neurosci 2003 23 297 302 12514227 10.1523/JNEUROSCI.23-01-00297.2003 PMC6742135

[B18] Ashdown H Dumont Y Ng M Poole S Boksa P Luheshi GN The role of cytokines in mediating effects of prenatal infection on the fetus: implications for schizophrenia Mol Psychiatry 2006 11 47 55 10.1038/sj.mp.4001748 16189509

[B19] Smith SE Li J Garbett K Mirnics K Patterson PH Maternal immune activation alters fetal brain development through interleukin-6 J Neurosci 2007 27 10695 10702 10.1523/JNEUROSCI.2178-07.2007 17913903 PMC2387067

[B20] Ponzio NM Servatius R Beck K Marzouk A Kreider T Cytokine levels during pregnancy influence immunological profiles and neurobehavioral patterns of the offspring Ann N Y Acad Sci 2007 1107 118 128 10.1196/annals.1381.013 17804539

[B21] Samuelsson AM Jennische E Hansson HA Holmäng A Prenatal exposure to interleukin-6 results in inflammatory neurodegeneration in hippocampus with NMDA/GABA_A _dysregulation and impaired spatial learning Am J Physiol Regul Integr Comp Physiol 2006 290 R1345 R1356 16357100 10.1152/ajpregu.00268.2005

[B22] Zaretsky MV Alexander JM Byrd W Bawdon RE Transfer of inflammatory cytokines across the placenta Obstet Gynecol 2004 103 546 550 10.1097/01.AOG.0000114980.40445.83 14990420

[B23] Yeargin-Allsopp M Rice C Karapurkar T Doernberg N Boyle C Murphy C Prevalence of autism in a US metropolitan area JAMA 2003 289 49 55 10.1001/jama.289.1.49 12503976

[B24] Palmer GW Claman HN Pregnancy and immunology: selected aspects Ann Allergy Asthma Immunol 2002 89 350 360 428 10.1016/S1081-1206(10)62034-0 12392378

[B25] Trowsdale J Betz AG Mother's little helpers: mechanisms of maternal-fetal tolerance Nat Immunol 2006 7 241 246 16482172 10.1038/ni1317

[B26] Denney JM Nelson EL Wadhwa PD Waters TP Mathew L Chung EK Goldenberg RL Culhane JF Longitudinal modulation of immune system cytokine profile during pregnancy Cytokine 2011 53 170 177 10.1016/j.cyto.2010.11.005 21123081 PMC4610033

[B27] Curry AE Vogel I Skogstrand K Drews C Schendel DE Flanders WD Hougaard DM Thorsen P Maternal plasma cytokines in early- and mid-gestation of normal human pregnancy and their association with maternal factors J Reprod Immunol 2008 77 152 160 10.1016/j.jri.2007.06.051 17692390

[B28] Wegmann TG Lin H Guilbert L Mosmann TR Bidirectional cytokine interactions in the maternal-fetal relationship: is successful pregnancy a TH2 phenomenon? Immunol Today 1993 14 353 356 10.1016/0167-5699(93)90235-D 8363725

[B29] Szekeres-Bartho J Halasz M Palkovics T Progesterone in pregnancy; receptor-ligand interaction and signaling pathways J Reprod Immunol 2009 83 60 64 10.1016/j.jri.2009.06.262 19880194

[B30] Raghupathy R Kalinka J Cytokine imbalance in pregnancy complications and its modulation Front Biosci 2008 13 985 994 10.2741/2737 17981605

[B31] Coe CL Lubach GR Prenatal origins of individual variation in behavior and immunity Neurosci Biobehav Rev 2005 29 39 49 10.1016/j.neubiorev.2004.11.003 15652253

[B32] Meyer U Yee BK Feldon J The neurodevelopmental impact of prenatal infections at different times of pregnancy: the earlier the worse? Neuroscientist 2007 13 241 256 10.1177/1073858406296401 17519367

[B33] Brown AS Derkits EJ Prenatal infection and schizophrenia: a review of epidemiologic and translational studies Am J Psychiatry 2010 167 261 280 10.1176/appi.ajp.2009.09030361 20123911 PMC3652286

[B34] Atladóttir HO Thorsen P Østergaard L Schendel DE Lemcke S Abdallah M Parner ET Maternal infection requiring hospitalization during pregnancy and autism spectrum disorders J Autism Dev Disord 2010 40 1423 1430 10.1007/s10803-010-1006-y 20414802

[B35] Shi L Smith SE Malkova N Tse D Su Y Patterson PH Activation of the maternal immune system alters cerebellar development in the offspring Brain Behav Immun 2009 23 116 123 10.1016/j.bbi.2008.07.012 18755264 PMC2614890

[B36] Golan HM Lev V Hallak M Sorokin Y Huleihel M Specific neurodevelopmental damage in mice offspring following maternal inflammation during pregnancy Neuropharmacology 2005 48 903 917 10.1016/j.neuropharm.2004.12.023 15829260

[B37] Gilmore JH Jarskog LF Vadlamudi S Maternal poly I:C exposure during pregnancy regulates TNFα, BDNF, and NGF expression in neonatal brain and the maternal-fetal unit of the rat J Neuroimmunol 2005 159 106 112 10.1016/j.jneuroim.2004.10.008 15652408

[B38] Laresgoiti-Servitje E Gómez-López N Olson DM An immunological insight into the origins of pre-eclampsia Hum Reprod Update 2010 16 510 524 10.1093/humupd/dmq007 20388637

[B39] Lash GE Robson SC Bulmer JN Review: Functional role of uterine natural killer (uNK) cells in human early pregnancy decidua Placenta 2010 31 Suppl S87 S92 20061017 10.1016/j.placenta.2009.12.022

[B40] Grether JK Croen LA Anderson MC Nelson KB Yolken RH Neonatally measured immunoglobulins and risk of autism Autism Res 2010 3 323 332 10.1002/aur.160 21182209

[B41] Cho SH Stanciu LA Holgate ST Johnston SL Increased interleukin-4, interleukin-5, and interferon-γ in airway CD4^+ ^and CD8^+ ^T cells in atopic asthma Am J Respir Crit Care Med 2005 171 224 230 15502111 10.1164/rccm.200310-1416OC

[B42] Magnan AO Mély LG Camilla CA Badier MM Montero-Julian FA Guillot CM Casano BB Prato SJ Fert V Bongrand P Vervloet D Assessment of the Th1/Th2 paradigm in whole blood in atopy and asthma: increased IFN-γ-producing CD8^+ ^T cells in asthma Am J Respir Crit Care Med 2000 161 1790 1796 10852746 10.1164/ajrccm.161.6.9906130

[B43] Tamási L Bohács A Pállinger E Falus A Rigó J Jr Müller V Komlósi Z Magyar P Losonczy G Increased interferon-γ- and interleukin-4-synthesizing subsets of circulating T lymphocytes in pregnant asthmatics Clin Exp Allergy 2005 35 1197 1203 10.1111/j.1365-2222.2005.02322.x 16164448

[B44] Kumar RK Webb DC Herbert C Foster PS Interferon-γ as a possible target in chronic asthma Inflamm Allergy Drug Targets 2006 5 253 256 10.2174/187152806779010909 17168796

[B45] Ngoc PL Gold DR Tzianabos AO Weiss ST Celedón JC Cytokines, allergy, and asthma Curr Opin Allergy Clin Immunol 2005 5 161 166 10.1097/01.all.0000162309.97480.45 15764907

[B46] Bauer S Kerr BJ Patterson PH The neuropoietic cytokine family in development, plasticity, disease and injury Nat Rev Neurosci 2007 8 221 232 17311007 10.1038/nrn2054

[B47] Boksa P Effects of prenatal infection on brain development and behavior: a review of findings from animal models Brain Behav Immun 2010 24 881 897 10.1016/j.bbi.2010.03.005 20230889

[B48] Patterson PH Immune involvement in schizophrenia and autism: etiology, pathology and animal models Behav Brain Res 2009 204 313 321 10.1016/j.bbr.2008.12.016 19136031

[B49] Carson MJ Doose JM Melchior B Schmid CD Ploix CC CNS immune privilege: hiding in plain sight Immunol Rev 2006 213 48 65 10.1111/j.1600-065X.2006.00441.x 16972896 PMC2633103

[B50] Fricchione G Daly R Rogers MP Stefano GB Neuroimmunologic influences in neuropsychiatric and psychophysiologic disorders Acta Pharmacol Sin 2001 22 577 587 11749820

[B51] Zhu Y Yu T Zhang XC Nagasawa T Wu JY Rao Y Role of the chemokine SDF-1 as the meningeal attractant for embryonic cerebellar neurons Nat Neurosci 2002 5 719 720 12080344 10.1038/nn881 PMC2072873

[B52] Rostene W Kitabgi P Parsadaniantz SM Chemokines: a new class of neuromodulator? Nat Rev Neurosci 2007 8 895 903 10.1038/nrn2255 17948033

[B53] Bondy SC Campbell A Developmental neurotoxicology J Neurosci Res 2005 81 605 612 10.1002/jnr.20589 16035107

[B54] Myren M Mose T Mathiesen L Knudsen LE The human placenta: an alternative for studying foetal exposure Toxicol In Vitro 2007 21 1332 1340 10.1016/j.tiv.2007.05.011 17624715

[B55] Simister NE Placental transport of immunoglobulin G Vaccine 2003 21 3365 3369 10.1016/S0264-410X(03)00334-7 12850341

[B56] Hauguel-de Mouzon S Guerre-Millo M The placenta cytokine network and inflammatory signals Placenta 2006 27 794 798 10.1016/j.placenta.2005.08.009 16242770

[B57] Murphy SP Tayade C Ashkar AA Hatta K Zhang J Croy BA Interferon γ in successful pregnancies Biol Reprod 2009 80 848 859 10.1095/biolreprod.108.073353 19164174 PMC2849832

