# Supplementary material for: Increased midgestational IFN-γ, IL-4 and IL-5 in women bearing a child with autism: A case-control study
Source: Mol Autism. 2011 Aug 2;2:13. doi: 10.1186/2040-2392-2-13 (PMC3170586; doi:10.1186/2040-2392-2-13)
Supplement: Additional file 1 — Adjusted odds ratios for covariates. Adjusted odds ratios for each covariate analyzed in the subject population for selected analytes from Table 3. [file 2040-2392-2-13-S1.DOC]

| **Additional File 1.** | | | | | | | | | | |  |  |  |  |  |
| --- | --- | --- | --- | --- | --- | --- | --- | --- | --- | --- | --- | --- | --- | --- | --- |
|  |  |  |  |  |  |  |  |  |  |  |  |  |  |  |  |
| **Mothers of ASD vs. Mothers of GP** | |  |  |  |  |  |  |  |  |  |  |  |  |  |  |
|  | **IFN-γ** | | | **IL-2** | | | **IL-4** | | | **IL-5** | | | **IL-6** | | |
|  | **OR_adj_** | **95% CI** | | **OR_adj_** | **95% CI** | | **OR_adj_** | **95% CI** | | **OR_adj_** | **95% CI** | | **OR_adj_** | **95% CI** | |
| log of Analyte | **1.52** | **1.19** | **1.93** | 1.22 | 0.96 | 1.57 | **1.51** | **1.12** | **2.03** | **1.45** | **1.07** | **1.98** | 1.10 | 0.97 | 1.26 |
| Maternal Age (years) | **1.11** | **1.03** | **1.19** | **1.10** | **1.03** | **1.18** | **1.10** | **1.03** | **1.18** | **1.10** | **1.03** | **1.18** | **1.11** | **1.04** | **1.18** |
| Maternal Race (Asian vs. White) | 0.58 | 0.19 | 1.79 | 0.54 | 0.18 | 1.58 | 0.55 | 0.18 | 1.66 | 0.54 | 0.18 | 1.62 | 0.55 | 0.18 | 1.64 |
| Maternal Race (Other vs. White) | 0.83 | 0.14 | 4.80 | 1.19 | 0.22 | 6.36 | 0.94 | 0.17 | 5.28 | 0.96 | 0.17 | 5.31 | 1.22 | 0.24 | 6.32 |
| Ethnicity (Hispanic vs Non-Hispanic) | 1.48 | 0.47 | 4.65 | 1.69 | 0.55 | 5.16 | 1.71 | 0.55 | 5.33 | 1.62 | 0.53 | 4.95 | 1.60 | 0.53 | 4.83 |
| Country of Birth (Mexico vs US) | **0.23** | **0.07** | **0.81** | **0.21** | **0.06** | **0.71** | **0.19** | **0.06** | **0.66** | **0.20** | **0.06** | **0.68** | **0.21** | **0.06** | **0.71** |
| Country of Birth (Other vs US) | 2.43 | 0.85 | 6.96 | 2.11 | 0.77 | 5.84 | 2.18 | 0.77 | 6.17 | 2.19 | 0.78 | 6.16 | 1.88 | 0.68 | 5.17 |
| GA at time of blood draw (weeks) | 1.04 | 0.99 | 1.08 | 1.03 | 0.99 | 1.08 | 1.04 | 1.00 | 1.08 | 1.04 | 1.00 | 1.08 | 1.04 | 1.00 | 1.08 |
| Maternal Weight (lbs) | 1.01 | 0.99 | 1.02 | 1.00 | 0.99 | 1.01 | 1.00 | 0.99 | 1.02 | 1.00 | 0.99 | 1.01 | 1.00 | 0.99 | 1.01 |
|  |  |  |  |  |  |  |  |  |  |  |  |  |  |  |  |
|  |  |  |  |  |  |  |  |  |  |  |  |  |  |  |  |
| **Mothers of ASD vs. Mothers of DD** | |  |  |  |  |  |  |  |  |  |  |  |  |  |  |
|  | **IFN-γ** | | | **IL-2** | | | **IL-4** | | | **IL-5** | | | **IL-6** | | |
|  | **OR_adj_** | **95% CI** | | **OR_adj_** | **95% CI** | | **OR_adj_** | **95% CI** | | **OR_adj_** | **95% CI** | | **OR_adj_** | **95% CI** | |
| log of Analyte | 1.46 | 0.94 | 2.26 | 1.31 | 0.77 | 2.21 | 1.19 | 0.70 | 2.03 | 1.70 | 0.87 | 3.34 | 0.79 | 0.62 | 1.02 |
| Maternal Age (years) | 1.07 | 0.94 | 1.21 | 1.07 | 0.94 | 1.21 | 1.08 | 0.95 | 1.22 | 1.09 | 0.96 | 1.24 | 1.12 | 0.98 | 1.28 |
| Maternal Race (Asian vs. White) | 0.10 | 0.00 | 2.93 | 0.13 | 0.00 | 4.58 | 0.17 | 0.01 | 5.66 | 0.18 | 0.01 | 5.76 | 0.25 | 0.01 | 8.10 |
| Maternal Race (Other vs. White) | 0.06 | 0.00 | 1.20 | 0.08 | 0.00 | 1.76 | 0.09 | 0.00 | 1.94 | 0.10 | 0.00 | 2.12 | 0.12 | 0.01 | 2.35 |
| Ethnicity (Hispanic vs Non-Hispanic) | 0.49 | 0.05 | 4.60 | 0.49 | 0.05 | 4.67 | 0.57 | 0.06 | 5.24 | 0.61 | 0.07 | 5.00 | 0.92 | 0.09 | 9.61 |
| Country of Birth (Mexico vs US) | **0.08** | **0.01** | **0.74** | **0.09** | **0.01** | **0.76** | **0.10** | **0.01** | **0.84** | **0.08** | **0.01** | **0.72** | **0.10** | **0.01** | **0.93** |
| Country of Birth (Other vs US) | 4.00 | 0.27 | 58.41 | 3.36 | 0.20 | 56.11 | 3.31 | 0.19 | 57.85 | 2.66 | 0.15 | 46.42 | 3.04 | 0.19 | 49.43 |
| GA at time of blood draw (weeks) | 1.04 | 0.95 | 1.13 | 1.05 | 0.96 | 1.14 | 1.05 | 0.97 | 1.14 | 1.06 | 0.98 | 1.15 | 1.07 | 0.98 | 1.17 |
| Maternal Weight (lbs) | 1.00 | 0.98 | 1.02 | 1.00 | 0.97 | 1.02 | 1.00 | 0.98 | 1.02 | 1.00 | 0.98 | 1.02 | 1.00 | 0.98 | 1.03 |
|  |  |  |  |  |  |  |  |  |  |  |  |  |  |  |  |
|  |  |  |  |  |  |  |  |  |  |  |  |  |  |  |  |
| **Mothers of DD vs. Mothers of GP** | |  |  |  |  |  |  |  |  |  |  |  |  |  |  |
|  | **IFN-γ** | | | **IL-2** | | | **IL-4** | | | **IL-5** | | | **IL-6** | | |
|  | **OR_adj_** | **95% CI** | | **OR_adj_** | **95% CI** | | **OR_adj_** | **95% CI** | | **OR_adj_** | **95% CI** | | **OR_adj_** | **95% CI** | |
| log of Analyte | 1.42 | 0.99 | 2.05 | **1.72** | **1.12** | **2.64** | **2.18** | **1.24** | **3.85** | 1.25 | 0.72 | 2.18 | **1.22** | **1.01** | **1.48** |
| Maternal Age (years) | 1.09 | 0.99 | 1.19 | 1.07 | 0.98 | 1.18 | 1.09 | 0.99 | 1.19 | 1.08 | 0.98 | 1.18 | 1.08 | 0.98 | 1.18 |
| Maternal Race (Asian vs. White) | 2.10 | 0.18 | 24.70 | 1.26 | 0.12 | 13.39 | 2.83 | 0.25 | 31.53 | 1.55 | 0.15 | 15.97 | 1.41 | 0.14 | 14.31 |
| Maternal Race (Other vs. White) | 4.58 | 0.40 | 52.75 | 5.18 | 0.48 | 55.40 | 7.14 | 0.67 | 75.71 | 5.94 | 0.56 | 62.94 | 4.02 | 0.37 | 43.51 |
| Ethnicity (Hispanic vs Non-Hispanic) | 1.52 | 0.29 | 7.86 | 1.45 | 0.28 | 7.57 | 2.07 | 0.38 | 11.28 | 1.55 | 0.29 | 8.21 | 1.52 | 0.28 | 8.27 |
| Country of Birth (Mexico vs US) | 1.62 | 0.36 | 7.20 | 1.63 | 0.36 | 7.51 | 1.14 | 0.24 | 5.46 | 1.75 | 0.39 | 7.76 | 1.62 | 0.35 | 7.56 |
| Country of Birth (Other vs US) | 1.31 | 0.16 | 10.56 | 1.66 | 0.21 | 12.82 | 1.01 | 0.14 | 7.42 | 1.33 | 0.18 | 9.52 | 1.55 | 0.22 | 11.16 |
| GA at time of blood draw (weeks) | 0.97 | 0.91 | 1.04 | 0.98 | 0.92 | 1.04 | 0.97 | 0.91 | 1.04 | 0.99 | 0.93 | 1.05 | 0.99 | 0.93 | 1.05 |
| Maternal Weight (lbs) | 1.00 | 0.99 | 1.02 | 1.00 | 0.99 | 1.02 | 1.00 | 0.99 | 1.02 | 1.00 | 0.99 | 1.01 | 1.00 | 0.99 | 1.01 |
