# Supplementary material for: Increased midgestational IFN-γ, IL-4 and IL-5 in women bearing a child with autism: A case-control study
Source: Mol Autism. 2011 Aug 2;2:13. doi: 10.1186/2040-2392-2-13 (PMC3170586; doi:10.1186/2040-2392-2-13)
Supplement: Additional file 2 — Crude odds ratios. Crude unadjusted odds ratios for all analytes in Table 3. [file 2040-2392-2-13-S2.DOC]

| **Additional File 2** | | | | | | |  |  |  |
| --- | --- | --- | --- | --- | --- | --- | --- | --- | --- |
| **Analyte** | **Mothers of ASD vs. Mothers of GP** | | | **Mothers of ASD vs. Mothers of DD** | | | **Mothers of DD vs. Mothers of GP** | | |
|  | **OR_crude_** | **95% CI** | | **OR_crude_** | **95% CI** | | **OR_crude_** | **95% CI** | |
| **GM-CSF** | 1.05 | 0.89 | 1.24 | **0.70** | **0.51** | **0.96** | 1.32 | 0.99 | 1.75 |
| **IFN-γ** | **1.39** | **1.13** | **1.70** | 1.15 | 0.84 | 1.57 | 1.30 | 0.96 | 1.77 |
| **IL-10** | 1.25 | 0.96 | 1.64 | 0.88 | 0.58 | 1.33 | **1.61** | **1.01** | **2.55** |
| **IL-12** | 0.90 | 0.60 | 1.36 | 0.77 | 0.36 | 1.66 | 0.94 | 0.54 | 1.65 |
| **IL-1b** | 0.99 | 0.85 | 1.15 | 0.81 | 0.64 | 1.03 | 1.10 | 0.88 | 1.38 |
| **IL-2** | 1.19 | 0.96 | 1.48 | 0.98 | 0.71 | 1.36 | **1.58** | **1.09** | **2.30** |
| **IL-4** | **1.36** | **1.06** | **1.75** | 0.94 | 0.65 | 1.36 | **1.93** | **1.21** | **3.08** |
| **IL-5** | **1.34** | **1.02** | **1.77** | 1.10 | 0.71 | 1.71 | 1.24 | 0.76 | 2.02 |
| **IL-6** | 1.08 | 0.96 | 1.22 | **0.79** | **0.65** | **0.95** | **1.26** | **1.06** | **1.52** |
| **TNF-a** | 1.02 | 0.83 | 1.24 | **0.65** | **0.46** | **0.91** | 1.34 | 0.99 | 1.82 |
| **IL-8** | 1.00 | 0.86 | 1.17 | 0.83 | 0.65 | 1.06 | 1.18 | 0.94 | 1.49 |
| **Eotaxin** | 1.18 | 0.82 | 1.68 | 0.90 | 0.52 | 1.57 | 1.19 | 0.63 | 2.23 |
| **IP-10** | 0.97 | 0.66 | 1.40 | 0.84 | 0.46 | 1.56 | 0.82 | 0.49 | 1.36 |
| **MCP-1** | 1.11 | 0.81 | 1.50 | 0.67 | 0.42 | 1.08 | 1.31 | 0.88 | 1.96 |
| **MIP-1a** | 1.06 | 0.92 | 1.24 | 0.81 | 0.62 | 1.07 | 1.18 | 0.94 | 1.49 |
| **MIP-1b** | 1.05 | 0.90 | 1.22 | 0.74 | 0.55 | 1.00 | 1.21 | 0.94 | 1.55 |
| **RANTES** | 0.94 | 0.69 | 1.28 | 0.85 | 0.47 | 1.54 | 1.05 | 0.61 | 1.78 |
