# Supplementary material for: Increased midgestational IFN-γ, IL-4 and IL-5 in women bearing a child with autism: A case-control study
Source: Mol Autism. 2011 Aug 2;2:13. doi: 10.1186/2040-2392-2-13 (PMC3170586; doi:10.1186/2040-2392-2-13)
Supplement: Additional file 3 — Limit of detection. Numbers and percentages of subjects found to be below the limit of detection (<LOD) for each analyte across groups. [file 2040-2392-2-13-S3.DOC]

**Additional File 3**

| **Cytokine** | **LOD** | **Autism, n=84** | | **DD, n=47** | | **GP, n=151** | |
| --- | --- | --- | --- | --- | --- | --- | --- |
|  |  | **N <LOD** | **% <LOD** | **N <LOD** | **% <LOD** | **N <LOD** | **% <LOD** |
| **GM-CSF** | 0.88 | 7 | 8.33 | 0 | 0.00 | 14 | 9.27 |
| **IFN-g** | 0.58 | 23 | 27.38* | 13 | 28.26* | 66 | 43.71 |
| **IL-10** | 3.40 | 43 | 51.19 | 26 | 56.52 | 84 | 55.63 |
| **IL-12** | 2.74 | 80 | 95.24 | 44 | 95.65 | 141 | 93.38 |
| **IL-1b** | 2.50 | 13 | 15.48 | 4 | 8.70 | 23 | 15.23 |
| **IL-2** | 1.45 | 47 | 55.95 | 19 | 41.30 | 94 | 62.25 |
| **IL-4** | 2.21 | 42 | 50.00 | 26 | 56.52 | 92 | 60.93 |
| **IL-5** | 1.75 | 42 | 50.00 | 27 | 58.70 | 83 | 54.97 |
| **IL-6** | 1.88 | 2 | 2.38 | 1 | 2.17 | 4 | 2.65 |
| **TNF-a** | 3.96 | 12 | 14.29 | 3 | 6.52 | 15 | 9.93 |
| **IL-8** | 6.23 | 0 | 0.00 | 3 | 6.52 | 2 | 1.32 |
| **Eotaxin** | 1.37 | 3 | 3.57 | 2 | 4.35 | 12 | 7.95 |
| **IP-10** | 2.01 | 22 | 26.19 | 8 | 17.39 | 43 | 28.48 |
| **MCP-1** | 13.03 | 1 | 1.19 | 0 | 0.00 | 4 | 2.65 |
| **MIP-1a** | 5.30 | 6 | 7.14 | 0 | 0.00 | 20 | 13.25 |
| **MIP-1b** | 6.10 | 9 | 10.71 | 1 | 2.17 | 18 | 11.92 |
| **RANTES** | 22.36 | 0 | 0.00 | 0 | 0.00 | 1 | 0.66 |
